# Plastid phylogenomics uncovers multiple species in *Medicago truncatula* (Fabaceae) germplasm accessions

**DOI:** 10.1038/s41598-022-25381-1

**Published:** 2022-12-07

**Authors:** In-Su Choi, Martin F. Wojciechowski, Kelly P. Steele, Andrew Hopkins, Tracey A. Ruhlman, Robert K. Jansen

**Affiliations:** 1 Department of Integrative Biology, University of Texas at Austin, Austin, TX 78712 USA; 2 School of Life Sciences, Arizona State University, Tempe, AZ 85287 USA; 3 Department of Biological Sciences and Biotechnology, Hannam University, Daejeon, 34054 Korea; 4 Division of Applied Science and Mathematics, Arizona State University, Mesa, AZ 85212 USA

**Keywords:** Phylogenetics, Genome

## Abstract

*Medicago truncatula* is a model legume that has been extensively investigated in diverse subdisciplines of plant science. *Medicago littoralis* can interbreed with *M. truncatula* and *M. italica*; these three closely related species form a clade, i.e. TLI clade. Genetic studies have indicated that *M. truncatula* accessions are heterogeneous but their taxonomic identities have not been verified. To elucidate the phylogenetic position of diverse *M. truncatula* accessions within the genus, we assembled 54 plastid genomes (plastomes) using publicly available next-generation sequencing data and conducted phylogenetic analyses using maximum likelihood. Five accessions showed high levels of plastid DNA polymorphism. Three of these highly polymorphic accessions contained sequences from both *M. truncatula* and *M. littoralis.* Phylogenetic analyses of sequences placed some accessions closer to distantly related species suggesting misidentification of source material*.* Most accessions were placed within the TLI clade and maximally supported the interrelationships of three subclades. Two *Medicago* accessions were placed within a *M. italica* subclade of the TLI clade. Plastomes with a 45-kb (*rpl20*-*ycf1*) inversion were placed within the *M. littoralis* subclade. Our results suggest that the *M. truncatula* accession genome pool represents more than one species due to possible mistaken identities and gene flow among closely related species.

## Introduction

*Medicago* L. (Fabaceae, Papilionoideae, Trifolieae) comprises about 87 species, including the important forage crop *Medicago sativa* L., and the biological model species *Medicago truncatula* Gaertn^1^. The *M. truncatula* cultivar Jemalong was initially nominated as a model plant in 1990^2^. Later R108-1 (hereafter R108), an in vitro-selected derivative of ecotype 108-1, was selected as a preferable plant partner for *Rhizobium* nodulation studies since it has superior in vitro regeneration, transformation, and symbiotic properties^3^. At that time, it was also noted that R108 had distinct morphological features and contained a nuclear genome ~ 17% smaller than that of Jemalong^3,4^. So far, Jemalong A17 and R108 have been the most extensively used accessions for comparative functional genomics compared to other numerous accessions of *M. truncatula*^5,6^.

The *Medicago* HapMap project (https://medicagohapmap2.org; up-dated https://medicago.legumeinfo.org/) produced more than 300 inbred lines (mainly from *M. truncatula*) and sequenced those using next-generation sequencing (NGS) technology^7–12^. Branca et al.^7^ and Yoder et al.^8^ recognized that some accessions labeled as *M. truncatula* (including R108) are phylogenetically closer to *Medicago italica* (Mill.) Grande and/or *Medicago littoralis* Rohde ex Loisel. Additional genetically diverged accessions from *M. truncatula* were subsequently revealed by Stanton-Geddes et al.^9^ and Kang et al.^10^. Some of those accessions are now listed as *M. murex* Willd. and *M. turbinata* (L.) All. at the *Medicago* HapMap website (https://medicago.legumeinfo.org/tools/germplasm/). However, the taxonomic identity of some other accessions remains elusive. *Medicago* is well known for tangled interspecific phylogenetic relationships^13^ and includes multiple taxonomic continua, such as the *M. littoralis*-*M. truncatula* complex^14^. These two species are known to produce hybrids^14^. The fact that the former species has also interbred with *M. italica* [= *Medicago tornata* (L.) Mill]^15,16^ supports the idea that the complex should be extended^16,17^. Molecular phylogenetic analyses^8,13,18,19^ have shown a monophyletic group of the three species *M. truncatula*, *M. littorialis*, and *M. italica* (hereafter TLI clade) and close phylogenetic relationships along with other congeners in the “truncatula clade” [sensu Yoder et al.^8^] but there is no consensus about their relationships. There is also debate concerning the designation “*M. truncatula* subsp. *tricycla*”^1,20,21^.

Plastid genome (plastome) unit structure is usually conserved across the angiosperms as a quadripartite configuration—two single-copy regions and a large inverted repeat (IR)^22^. Early Fabaceae (legumes) plastome studies^23,24^ showed that a species-rich lineage (IR-lacking clade or IRLC) lost one copy of the canonical angiosperm IR. *Medicago* belongs to the IRLC^25–28^ together with many species that have the potential for the biparental inheritance of the plastome^29,30^. A comparative plastome analysis of Fabaceae in Saski et al.^31^ showed that a plastome of *M. truncatula* (AC093544; Jemalong A17) lacks rearrangement after IR loss. However, Gurdon and Maliga^32^ sequenced plastomes of multiple *M. truncatula* accessions, and discovered an ~ 45-kb inversion of the region from *rpl20* to *ycf1*, mediated by a short (11 bp) T/A inverted repeat, from several individuals (including the R108 accession) of “*M. truncatula* subsp. *tricycla*”. Recently, plastomes of dozens of *Medicago* species have been sequenced^33–37^ and have shown multiple additional rearrangements across the genus. *Medicago* exhibits high plastid sequence variation at interspecific, intraspecific and intraindividual levels^32–38^. A recent plastome study of Jiao et al.^37^ found that two of three *M. littoralis* accessions had the same inversion as R108 (*i.e.* the 45-kb inversion) and putative intraindividual structural (or assembly) variation from four *Medicago* species [*Medicago coronata* (L.) Bartal., *Medicago constricta* Durieu*, M. littoralis*, and *M. soleirolii* Duby]. Jiao et al.^37^ verified intraindividual inversion variation of a 16-kb plastid region between a small IR (~ 300 bp) in *M. soleirolii* by polymerase chain reaction (PCR). However, the reasons why *M. truncatula* shows intraspecific plastome structural variation and how it is related to *M. littoralis* remain equivocal.

Here, we assembled 54 plastomes and conducted phylogenetic analyses focusing on *M. truncatula* and its closely related species. We aimed to (1) resolve the phylogenetic positions of diverse accessions labeled as *M. truncatula* in at least one previous study, (2) clarify the cause of unusual intraspecific plastome structural variation in *M. truncatula*, and (3) explore the relationship of the scientifically crucial R108 accession to the Jemalong A17 accession.

## Results

### Plastid genome assembly completeness

**Figure 1:**
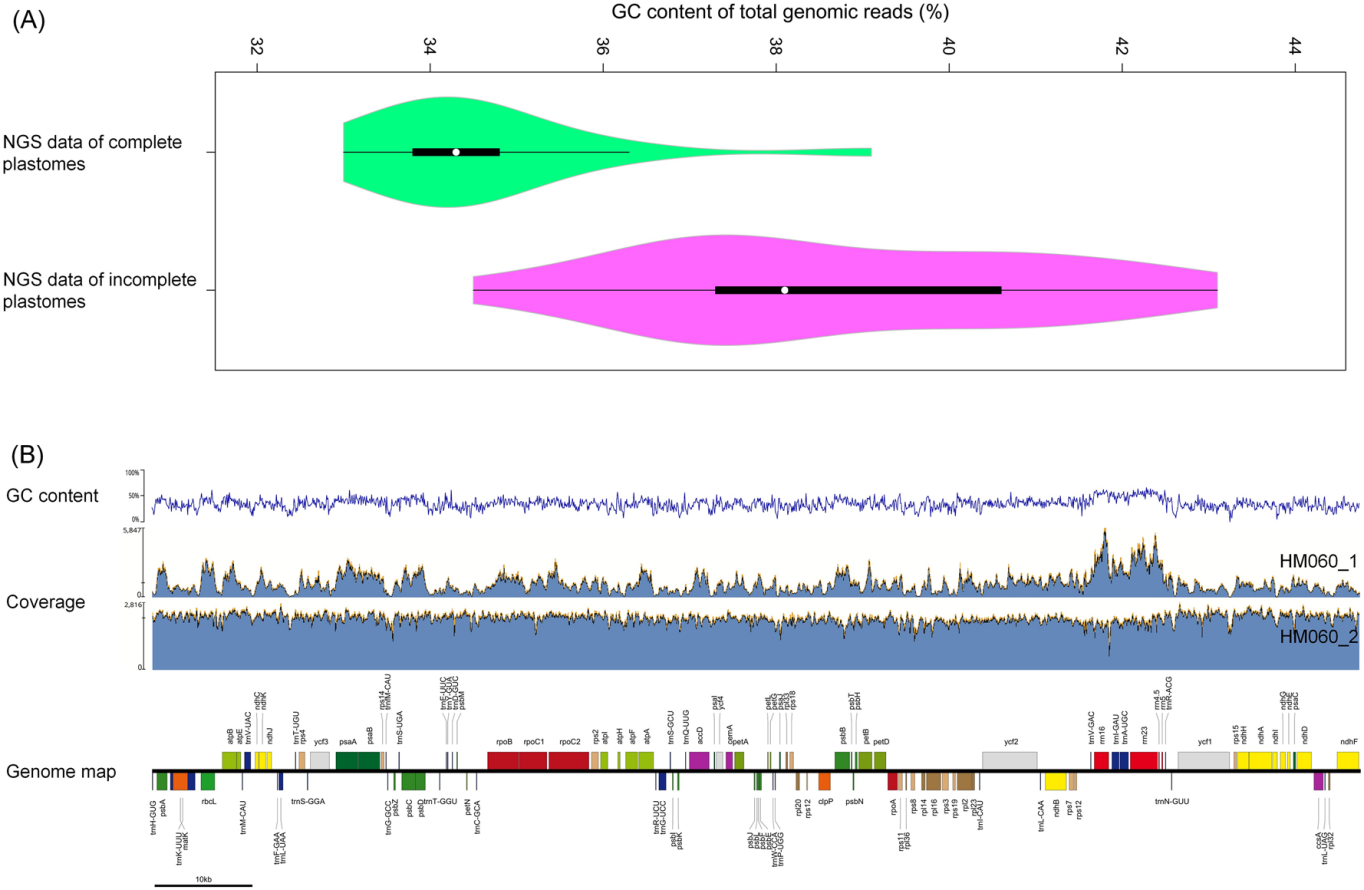
The relationship between GC content and plastome completeness. (**A**) Violin plot of the GC content between total genomic reads of complete and incomplete plastomes. (**B**) GC content fluctuation across the genome and corresponding changes of read coverage in HM060_1 (incomplete plastome) and HM060_2 (complete plastome).

Fifty-four plastomes of *M. truncatula* and related species accessions were assembled, 21 of which were completed and 33 were incomplete with gaps (Table S1). The GC content in raw NGS data files of completed plastomes varied from 33.0 to 39.1% while incomplete plastomes ranged 34.5 to 43.1% GC (Fig. 1A). The mean coverage varied from 159 × (HM253) to 16,040 × (HM030) (Table S1). Several incomplete plastomes showed coverage heterogeneity issues (excluding assembly gaps) relative to some of the complete plastomes. For example, read mapping results from HM060_1 showed coverage fluctuation, which appears to be positively correlated to GC content across the plastome (Fig. 1B). The length of complete plastomes ranged from 121,997 bp in HM274 to 124,339 bp in HM056_2. Polymorphic sites were observed in 20 plastomes, with five (HM018_1, HM029, HM261, HM292, and HM338) containing more than 50 variable sites (Table S1).

### Phylogenetic signal of *trnK*/*matK *sequences

**Figure 2:**
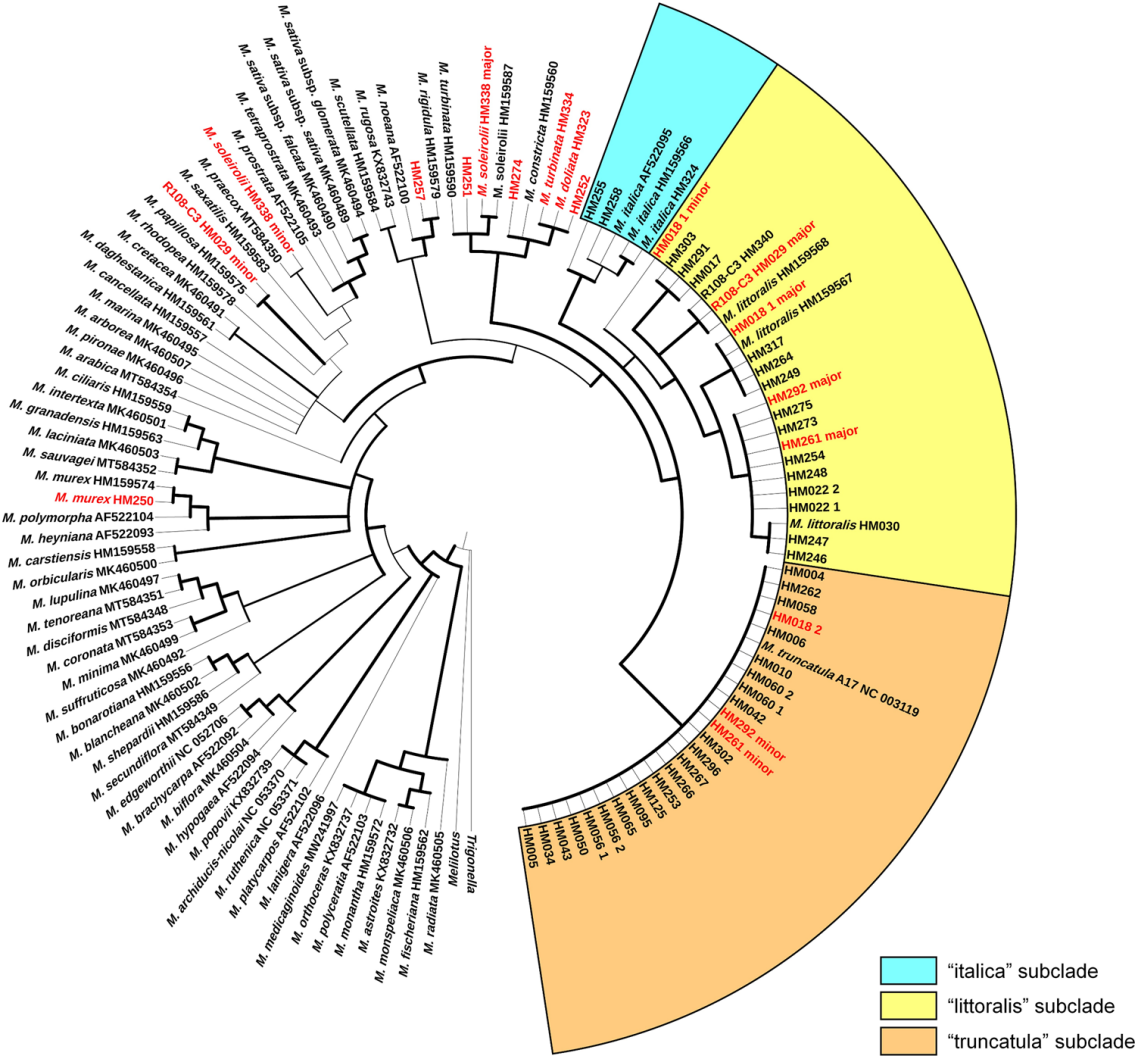
Maximum likelihood phylogeny of *Medicago* based on sequences of *trnK/matK* from *Medicago* (*M.*) species and two outgroup taxa. Members of the three subclades of the TLI clade are shaded with different colors. The wider branch width represents those lineages with higher bootstrap support values for each node. HapMap sequences placed outside the TLI clade or showing conflicting phylogenetic signals within a single NGS data or between first and second data from the same accession are indicated with red font.

The maximum likelihood (ML) tree of 126 *trnK* intron/*matK* gene sequences (including five minor variants from polymorphic plastomes) is shown in Fig. 2. Ten sequences from nine accessions (HM250, HM029_minor, HM338_minor, HM257, HM251, HM338_major, HM274, HM334, HM323, and HM252) were placed outside of the TLI clade. Hereafter, taxon identification from the *Medicago* HapMap website (https://medicago.legumeinfo.org/tools/germplasm/) is indicated in brackets following accession numbers. HM250 [*M. murex*] formed a monophyletic group with *M. murex* (HM159574). Two minor variants of HM029 [R108-C3] and HM338 [*M. soleirolii*], were more closely related to *M. papillosa* Boiss. and *M. praecox* DC., respectively. HM257 [*M. truncatula*] formed a monophyletic group with *M. rigidula* (L.) All. The major variant of HM338 [*M. soleirolii*] was found in the expected position close to HM159587 [*M. soleirolii*]. However, HM334 [*M. turbinata*] was not resolved as phylogenetically close to *M. turbinata* (HM159590) and formed a polytomy with HM323 [*M. doliata*] and HM252 [*M. turbinata*]. The positions of HM251 [*M. truncatula*] and HM274 [*M. truncatula*] were unresolved.

The rest of the sequences from newly generated plastomes were resolved within the TLI clade, divided into one of three subclades (the “italica”, “littoralis” and “truncatula” subclades), but phylogenetic relationships among the three subclades were not resolved. Three of the five highly polymorphic accessions showed mixed sequences of *M. truncatula* and *M. littoralis.* The minor variants of *M. truncatula* accessions HM261 and HM292 were part of the “truncatula” subclade while their major variants were found in the “littoralis” subclade. The two variants of HM018_1 were placed within the “littoralis” subclade but their polymorphic loci were permutable as a combination of sequences of *M. truncatula* Jemalong A17 (NC_003119) and *M. littoralis* (HM159567) (Fig. S1). A second NGS data file of HM018 (*i.e.* HM018_2) did not show a polymorphic signal and its *trnK*/*matK* sequence was identical to *M. truncatula* Jemalong A17 (NC_003119). Accessions HM255 [*M. murex*] and HM258 [*M. littoralis*] were placed within the “italica” subclade.

### Plastid phylogenomics and phylogenetic distribution of the 45-kb inversion

**Figure 3:**
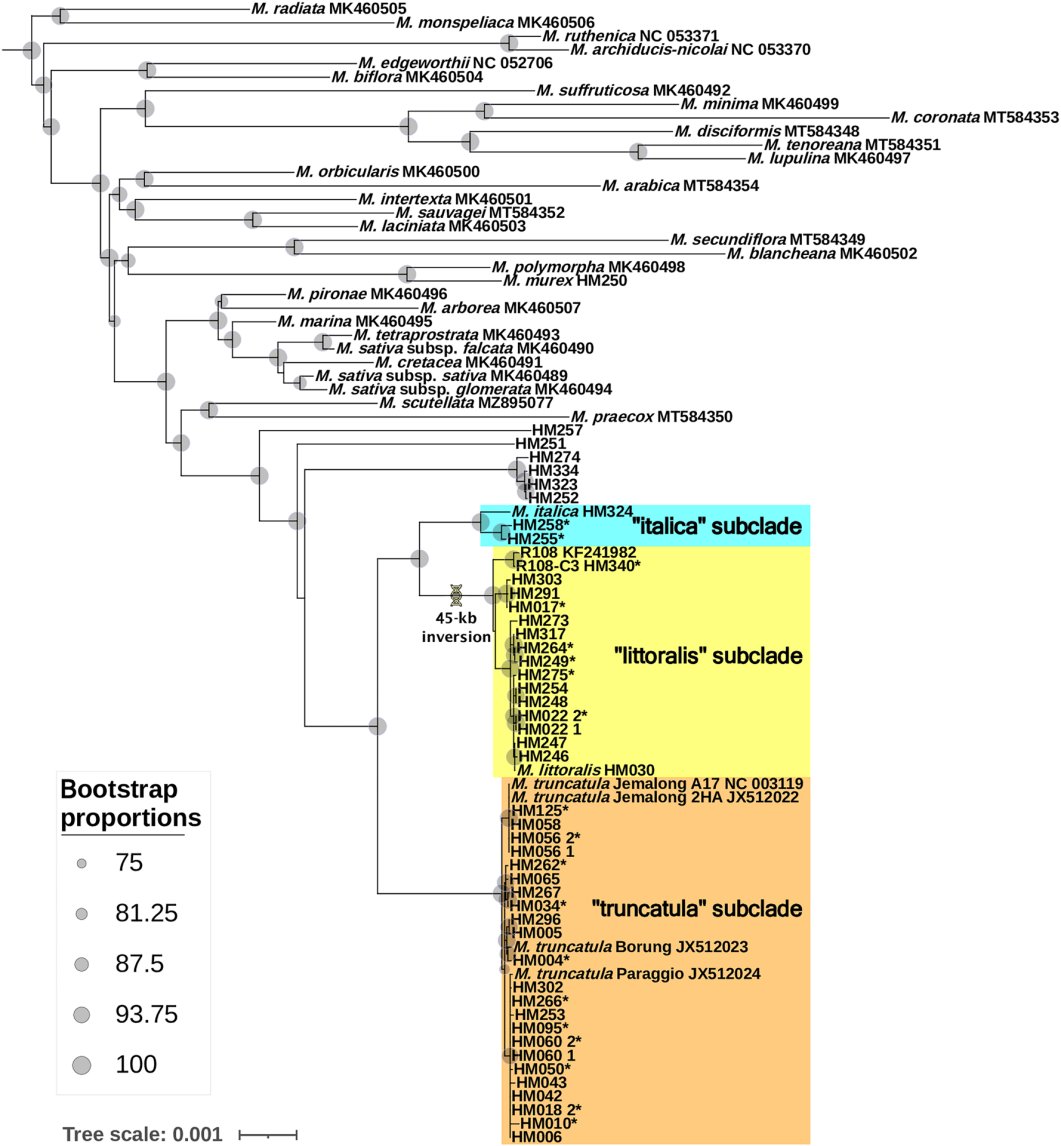
Phylogenetic relationship of *Medicago* (*M.*) species based on maximum likelihood analysis of 69 plastid coding sequences (CDSs). Note that outgroups and branches connecting those to the root node of the genus *Medicago* are omitted. Bootstrap support values (75–100%) for nodes are presented as grey circles of different sizes. Members of the three subclades within the TLI clade are shaded with different colors. Within the TLI clade, complete plastomes are marked with *. The scale indicates number of nucleotide substitutions per site.

**Figure 4:**
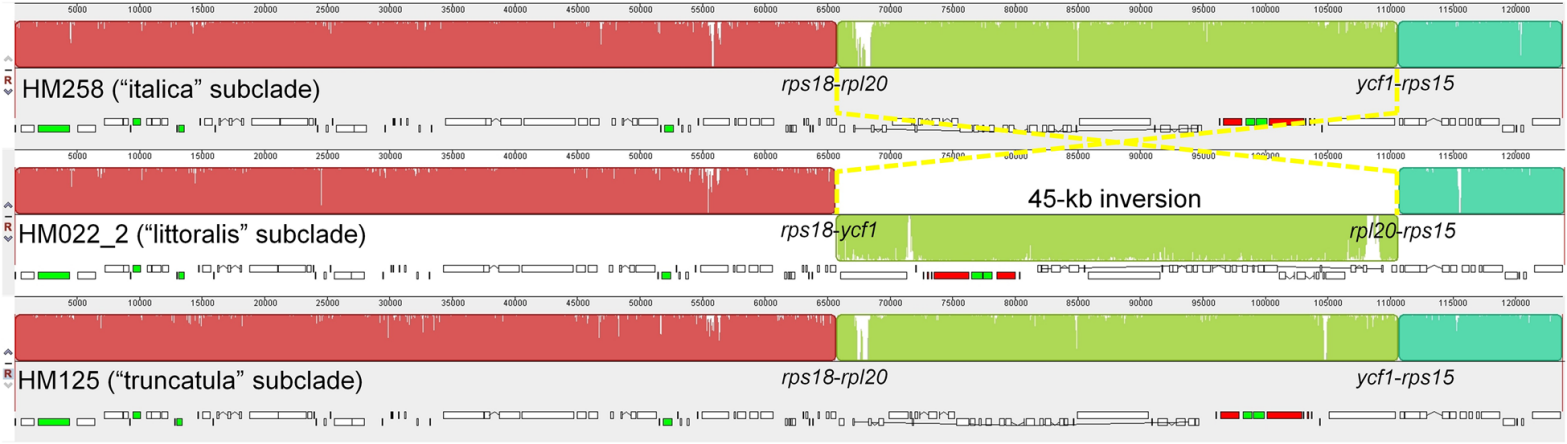
Mauve alignment of representative plastomes of the three subclades of the TLI clade. Alignment coordinates are given in bp above each sequence. Each locally collinear block (LCB) is differently colored, and histograms within it represent pairwise sequence identity. The inverted 45-kb LCB in the “littoralis” subclade is shown as a flipped block across the plane.

The concatenated length of the alignment of 69 CDSs (Table S2) was 51,883 bases, of which 2,921 were parsimony-informative sites. These 69 CDSs were recovered from all but five plastomes (Table S3). The plastid phylogenomic analysis resolved the monophyly of the TLI clade and each of the three subclades with maximal bootstrap support (BS = 100) values (Fig. 3). The analysis resolved the “truncatula” subclade as sister to a monophyletic group, comprised of the “italica” and “littoralis” subclades. In total, 17 plastomes from 16 *Medicago* accessions were placed within the “italica” (2) and “littoralis” (15) subclades. The 45-kb inversion (Fig. 4) was only found in six of the newly completed plastomes (HM340, HM017, HM264, HM249, HM275, and HM022_2; Table S1) and a previously sequenced R108 plastome (KF241982) within the “littoralis” subclade (Fig. 3). The remaining eight accessions of the “littoralis” subclade represent incomplete plastome assemblies and thus the presence of the 45 kb inversion could not be unambiguously determined (Table S1).

### Plastid *rps18*-*rpl20* sequences in the R108 nuclear genome

We found two instances of plastid sequences (*rps18*-*rpl20)* in the nuclear genome (NUPTs) that at least partly spanned loci on chromosome (Chr) 2 (position: 22,215,328–22,215,718) and Chr6 (position: 14,462,536–14,463,231) of the R108 nuclear genome (Fig. S2). The locus on Chr2 did not have a binding site for either PCR primer. The locus in Chr6 included one primer binding site of identical sequence compared to the plastome of *M. truncatula* Jemalong 2HA (JX512022).

## Discussion

Here we used massive quantities of NGS data generated by the *Medicago* HapMap project to explore plastid sequence diversity at interspecific, intraspecific, and intraindividual levels with particular attention to *M. truncatula* and related species. Some plastomes could not be completed due to the poor quality of some NGS data rather than quantity. We have analyzed the extraordinary heterogeneity of *M. truncatula* accessions in a systematic context and suggest explanations based on natural and artificial phenomena.

### Why did some plastomes fail to assemble from NGS data?

We completed plastomes from 21 NGS data files while we obtained only fragmented plastid contigs from the other 33 (Table S1). An obvious feature of accessions with incomplete, gapped plastomes was high GC% in total reads along with heterogeneous read coverage that matches GC% variation across the genome (see HM060_1 in Fig. 1B). Previous studies have indicated that GC bias in NGS data lowers assembly completeness^39^. It is also known that coverage heterogeneity can introduce plastome assembly gaps where *k*-mers do not overlap^40^. We tried to overcome the coverage heterogeneity issue by including a large number of raw NGS reads for assembly of HM030 (Table S1). Even though the mean coverage for HM030 plastid contigs was very high (16,040-fold), the plastome could not be completed. We also compared plastome completeness between assemblies from NGS data files of four other accessions (HM018, HM022, HM056, and HM060). While the first NGS data files of four accessions (HM018_1, HM022_1, HM056_1, and HM060_1) from earlier HapMap studies^7–10^ had higher GC content and produced incomplete plastomes, the second NGS data file of the same accessions (HM018_2, HM022_2, HM056_2, and HM060_2) from later studies^11,12^ showed lower GC% and successfully assembled complete plastomes with constant read coverage (for example, see HM060_2 in Fig. 1B).

The GC% differences across the HapMap NGS data are not likely caused by actual genomic differences but by the over-representation of GC-rich regions over AT-rich regions before or during NGS sequencing. These biases in NGS data are likely causes of plastome coverage heterogeneity and incompleteness issues in this study. A similar pattern of over-representation of GC-rich regions (especially around plastid rRNA genes) was observed in the heterotrophic gymnosperm *Parasitaxus usta* (Podocarpaceae)^41^. This was interpreted as uneven sequencing due to compositional-related genomic fragmentation biases. The *Medicago* HapMap project extracted DNA for NGS sequencing from ~ 30-day-old dark-grown seedlings^7–12^. Changes in light conditions can trigger plastid DNA replication as well as degradation in *M. truncatula*^42^. Flanking regions of the *rrn16* rRNA gene that showed high coverage are not just high GC sites (see Fig. 1B) but also the putative origins of replication (oriA and oriB)^42,43^. Many steps in NGS can introduce GC bias, but PCR during library preparation is a principal source^44^. It is likely that resampling or modification of NGS library preparation would be better options to overcome coverage heterogeneity issues than simply generating more data from the same library.

### Misidentification, plastid genome introgression and contamination

Plastid sequences of some *Medicago truncatula* HapMap accessions showed genetic divergence from those of the TLI clade and were grouped more closely with other *Medicago* species (Figs. 2, 3). Misidentifications of the original accessions may be an explanation. Steier et al.^45^ found several misidentifications and mixed seed collections among United States Department of Agriculture (USDA) accessions of species of *Medicago* section *Buceras*. Steele et al.^46^ found that about 30% of analyzed USDA accessions labeled as *Medicago* section *Medicago* taxa are incorrectly identified. Similarly, in another model plant species, *Arabidopsis thaliana* (L.) Heynh. (Brassicaceae), about 5% of accessions are misidentified^47^.

Taxon misidentification has long been a problem in *Medicago* studies^1,17^. A recent study by Choi et al.^36^ also found misidentification of *Medicago polymorpha* L. as *Medicago noeana* Boiss. from USDA accessions. The *M*. *littoralis* accession sampled in Jiao et al.^37^ lacks the 45-kb inversion^32^ and was placed in a clade with *M*. *truncatula* accessions may also be a misidentification. These studies and our results indicate that some original seed accessions of the *Medicago* HapMap project are misidentified, mislabeled or mixed with other species. For example, morphologically *M. turbinata* is commonly confused with *M. doliata*^1^. From the *trnK*/*matK* phylogeny (Fig. 2) produced in this study, HM334 [*M. turbinata*] did not group with a reference sequence of *M. turbinata* (HM159590) and was a part of a polytomy with HM323 [*M. doliata*]. Furthermore, both HM334 and HM323 showed little genetic divergence in earlier phylogenetic analyses of nuclear SNP data^8^.

The *Medicago* HapMap project developed more than 300 inbred lines by self-fertilization for at least three generations from original seed populations and sequenced DNA from a pool of seedlings per a single inbred line^7–12^. However, our study showed conflicting phylogenetic signals even within a single NGS data file and between the first and second NGS data files from a single HapMap accession (Fig. 2). Plastome-wide sequence heteroplasmy (existence of multiple versions of plastomes in a cell or individual)^48,49^ that are not caused by recent de novo mutations but by genetic contribution from multiple species is not likely to exist in inbred lines. However, genetic representation of both parents could arise in outcrossing descendants since *M. truncatula* has biparental inheritance of plastid DNA^50^. It is also possible that the sequence polymorphism detected in a single NGS file represents interindividual variation if three generations by self-fertilization were not sufficient to remove genetic variation from the original seed population.

In this study, NGS data of HM018_1 from earlier HapMap studies^7–10^ showed mixed *trnK*/*matK* sequences of the “truncatula” and “littoralis” subclades, while data of HM018_2 from later studies^11,12^ showed identical sequences to the “truncatula” subclade (Fig. 2, Fig. S1). HM018 was removed from the HapMap collection due to its unreliable identity (Nevin Young, University of Minnesota, personal communication). Our analyses suggest that some of the HapMap accessions are not “pure” inbred lines or became “contaminated” before or during the NGS sequencing process. Even though *M. truncatula* is highly self-fertile, it also employs an explosive tripping pollination system allowing for occasional insect-mediated outcrossing^1,51^. Furthermore, *M. truncatula* hybridizes with *M. littoralis*^14^ while the latter also hybridizes with *M. italica*^15^*,* enabling gene transfer among the three species^16^. Whether 16 HapMap accessions lacking high levels of plastid DNA polymorphism in the “italica” and “littoralis” subclades in our study (Fig. 3) represent misidentified plants (i.e. “pure” *M. italica* or *M. littoralis*) or introgressants remains uncertain due to possible plastid DNA captures. Also, the possibility of metadata labeling issues of NGS data that can mix different read sets cannot be ruled out (Andrew Farmer, National Center for Genome Resources, personal communication).

The importance of voucher specimens has been emphasized in molecular phylogenetic studies^52^. Linking scientific knowledge to a taxonomic group and reproducing experiments can be challenging without vouchers, especially for *Medicago* considering its’ interspecific hybridization and frequent misidentification of species. Pooling tissues from multiple individuals from a seed population is inadvisable. For an individual without enough tissue for an experimental material as well as a voucher specimen, a photo voucher can be an alternative choice^53^. Phylogenetic analysis of additional reliable reference sequences of nuclear DNA from voucher specimens with morphological diagnostic characters would allow correct species identification for these original seed populations and inbred lines of the *Medicago* HapMap project.

### Plastomes with 45-kb inversion belong to the “littoralis” subclade

The 45-kb plastome inversion between the *rpl20* and *ycf1* genes in the R108 accession and three out of seven individuals of “*Medicago truncatula* subsp. *tricycla*” was reported by Gurdon and Maliga in 2014^32^. Our study revealed that the plastomes with the inversion are considerably diverged in their CDS sequences from typical *M. truncatula* accessions without the inversion and form a monophyletic group (*i.e.* the “littoralis” subclade) sister to the “italica” but not the “truncatula” subclade (Fig. 4). This suggests that the occurrence of two distinct plastome configurations in *M. truncatula* accessions is different from that of intraspecific inversion isomers, which share little or no DNA sequence divergence but mainly differ with regard to their gene order due to highly active genome rearrangements, such as found in Pinaceae^54^, Cupressaceae^55,56^, *Taxus* (Taxaceae)^57^, *Eleocharis* (Cyperaceae)^58^, *Monsonia emarginata* (L.f.) L'Hér. (Geraniaceae)^59^, and *Medicago soleirolii* (Fabaceae)^37^. The 45-kb inversion also differs from homoplastic plastome structural evolution found among various distinct taxonomic groups in Papilionoideae^33,34,60–62^. Our data support the hypothesis that the 45-kb inversion initially occurred early in *M. littoralis* and may have recently introgressed into related taxa.

Gurdon and Maliga^32^ reported PCR amplification products for *rps18*-*rpl20* (no inversion) as well as *rps18*-*ycf1* (45-kb inversion) plastome configurations in a single DNA template (R108) and suggested the existence of NUPTs. We searched reference level chromosomal assemblies of the R108 nuclear genome^6^ and found two NUPT loci (Fig. S2), including the *rps18-rpl20* region but excluding one or both primers (58.549R and S7_57758F) binding sites of Gurdon and Maliga^32^. Amplification of NUPT loci of *rps18*-*rpl20* sequences by non-specific binding of primers is a less likely but possible explanation. Alternatively, as discussed earlier, the plastomes with and without the inversion can co-exist within a single individual due to possible plastid DNA introgression from or to *M. littoralis*.

### Systematic context of the R108 accession

Even before the use of *M. truncatula* as a model plant in 1990^2^, it was considered part of a species complex with *M. littoralis*^14^ that was extended to include *M. italica*^16,17^. Morphological analyses of members of this species complex^17,63,64^ demonstrated intermediate individuals (putative introgressants) of *M. truncatula*-*M. littoralis* and *M. littoralis*-*M. italica* as well as relatively pure representatives of each of these three species. The plastid *trnK*/*matK* phylogeny of Steele et al.^19^ did not resolve the phylogenetic relationship among the three species. However, our plastome-scale phylogeny resolved the relationships with maximal support as the “truncatula” subclade sister to a monophyletic group comprised of the “italica” and “littoralis” subclades (Fig. 3). This grouping is essentially the same topology based on nuclear SNP data from Yoder et al.^8^ and nuclear ribosomal internal transcribed spacer data from Bena^18^.

Along with the Jemalong A17 accession, R108 is the most frequently used accession in *M. truncatula* genomic research^5,6^. The R108 accession is often called “*M. truncatula* subsp. *tricycla*”, but without authorship^21^. The multiple names with the epithet “*tricycla*” were listed as synonyms of *M. italica* (= *Medicago tricycla* DC.), *M. littoralis* (= *Medicago tricycla* Senn.) and *M. truncatula* [= *M. truncatula* Gaertn. var. *tricycla* (Nègre) Heyn] in Small^1^. We could not find any formal taxonomic treatment for the designation “*M. truncatula* subsp. *tricycla*” leaving its taxonomic identity unclear. Apart from the ambiguity in taxonomic identity, Ellwood et al.^20^ analyzed microsatellite variation across 192 *M. truncatula* accessions and recognized 15 genetically distinct “*M. truncatula* subsp. *tricycla*” accessions as a paraphyletic grade. Small^1^ analyzed fruits of 11 “*M. truncatula* subsp. *tricycla*” accessions (out of the 15) from Ellwood et al.^20^ and concluded that all of these have a degree of *M. littoralis* genetic background, but with varying contributions from *M. truncatula* or *M. italica.* Gurdon and Maliga^32^ also showed that individuals of “*Medicago truncatula* subsp. *tricycla*” are genetically heterogeneous in plastome structure. These lines of evidence suggest that the concept of “*Medicago truncatula* subsp. *tricycla*” is not acceptable or valid, as previously concluded by Small^1^, and therefore should be abandoned. According to our current study, the R108 accession appears to have originated from explants of *M. littoralis* or at least its introgressant.

## Materials and methods

### Acquisition of previously generated next-generation sequencing data

We downloaded NGS data files of 50 *M. truncatula* and related species accessions (Table S1) previously generated by the *Medicago* HapMap project^7–12^ from the Sequence Read Archive (SRA) (https://www.ncbi.nlm.nih.gov/sra). Each of the NGS data files of 45 accessions includes a single SRA run, while a single data file of HM030 is a merger of seven SRA runs. Two separately obtained NGS SRA runs were available for four HapMap accessions (HM018, HM022, HM056, and HM060), so we downloaded both for each accession. We added a suffix (_1) for each of the four first NGS data files from earlier HapMap publications^7–10^. For the second NGS data file of the four from the later studies^11,12^, we added a different suffix (_2). In total, 54 data files were prepared for the downstream analyses. Our sampling included all HapMap accessions placed within the “truncatula” clade based on the molecular phylogeny of Yoder et al.^8^. We also included all 21 accessions that showed high divergence from most *M. truncatula* accessions in Stanton-Geddes et al.^9^. The seven HapMap accessions (HM253, HM261, HM262, HM266, HM267, HM273, and HM275) from the same seed populations of the seven “*M. truncatula* subsp. *tricycla*” plants in Gurdon and Maliga^32^ were also sampled in this study.

### Plastid genome assembly and *trnK*/*matK* phylogeny

Plastome sequences were assembled using GetOrganelle v1.7.4.1^40^. The quality, coverage, and sequence polymorphism of plastid assemblies were checked by read mapping using the low sensitivity option in Geneious Prime 2022.1.1 (https://www.geneious.com/). Plastome assemblies were annotated using the annotation of *Medicago radiata* L. (NC_042854.1) in Geneious Prime. A plastome map of HM060_2 was drawn using OGDRAW v. 1.3.1^65^. Polymorphic sites were counted from plastid loci with 100-fold minimum coverage and 0.25 minimum variant frequency using the “Find Variations/SNPs” function in Geneious Prime.

Sequences from plastomes of *Melilotus albus* Medik. (NC_041419) and *Trigonella foenum-graecum* L. (NC_042857) were sampled as outgroups. Sequences for the region including the *trnK* intron/*matK* gene were extracted from the plastome assemblies of the current study*.* Five of the accesions showed high levels of plastid DNA polymorphism, three of which showed mixed sequences of *M*. *truncatula* and *M*. *littoralis*. In addition, the *trnK*/*matK* sequences of Steele et al.^19^ and complete plastomes used in previous *Medicago* studies^33,34,66^ were included as ingroups (Table S4). Sequences were aligned using MAFFT v.7.450^67^, and the alignment algorithm was automatically selected. Maximum likelihood (ML) analysis based on a total of 126 *trnK*/*matK* sequences was conducted using IQ-TREE 2.2.0^68^ with 1000 bootstrap replications, and short branches (near-zero) were collapsed using the -czb option. A best-fit nucleotide substitution model was automatically selected based on the Bayesian Information Criterion.

For the convenience of downstream analyses, multiple plastid contigs from a single *Medicago* HapMap datafile were arranged as an incomplete plastome with gaps according to a reference complete plastome of the most phylogenetically close *Medicago* species shown from the *trnK*/*matK* ML tree or *M. truncatula* Jemalong A17 (NC_003119)*.*

### Plastid genome phylogeny and structural analysis

Among the 54 newly assembled plastomes, five with more than 50 polymorphic sites were excluded from the plastid phylogenomic analysis (Table S1). In addition, 37 plastomes from previous studies^32–34,66,69^ were included in the ingroup (Table S5). Sequences from plastomes of *Melilotus albus* (NC_041419) and *Trigonella foenum-graecum* (NC_042857) were sampled as outgroups. The 69 protein-coding sequences (CDSs) (Table S2), used in the phylogenomic analysis of *Medicago* by Choi et al.^33^, were extracted from plastomes and aligned as described above using MAFFT. A ML analysis was conducted as described above using IQ-TREE, which determined the best partition scheme. Visualization of phylogenetic trees were conducted using Interactive Tree Of Life (iTOL)^70^. The structure of each complete plastome was analyzed using genome alignment software Mauve 2.3.1^71^.

### Search for the plastid *rps18*-*rpl20* region in the R108 nuclear genome

Previously, Gurdon and Maliga^32^ proposed the existence of the plastid *rps18*-*rpl20* region in the nuclear genome of the R108 (*i.e.* NUPT)^72^ based on their PCR experiments. Here, we tried to verify *rps18*-*rpl20* NUPTs in silico. The up-to-date chromosomal-scale nuclear genome of the R108 (GWHBFSB00000000)^6^ was downloaded from the National Genomics Data Center (https://ngdc.cncb.ac.cn/?lang=en). A sequence search was conducted by BLAST^73^ implemented in Geneious Prime with default options of discontiguous MegaBLAST using the *rps18*-*rpl20* region of *M. truncatula* Jemalong 2HA (JX512022) as a query.

## Supplementary Information

Supplementary Figures.

Supplementary Tables.

## Data Availability

All plastid genomes, sequence alignments, and trees that were generated from this study are submitted to Dryad (https://doi.org/10.5061/dryad.tmpg4f51m).
