# Supplementary material for: Plastid phylogenomics uncovers multiple species in Medicago truncatula (Fabaceae) germplasm accessions
Source: Sci Rep. 2022 Dec 7;12:21172. doi: 10.1038/s41598-022-25381-1 (PMC9729603; doi:10.1038/s41598-022-25381-1)
Supplement: Supplementary file 1 — Supplementary Figures. [file 41598_2022_25381_MOESM1_ESM.pdf]

Accessions	Aligned position								
	190	490	879	1019	1023	1026	1498	1606	1932
M. italica _AF522095	G	A	A	G	T	T	T	G	G
M. italica _HM159566	G	A	A	T	T	T	T	G	G
M. littoralis _HM159568	A	C	A	G	A	T	T	G	G
M. littoralis _HM159567	A	C	A	G	A	G	T	G	A
HM018_1	A	C	A	G	A	G	T	G	A
	G	A	G	T	T	T	G	A	G
HM018_2	G	A	G	T	T	T	G	A	G
M. truncatula _A17_NC_003119	G	A	G	T	T	T	G	A	G

Figure S1. Plastid sequence (*trnK/matK*) polymorphism in HM018_1. Four reference sequences from the TLI clade and HM018_2 are included. Nine polymorphic sites of HM018_1 are presented. Major and minor variants in HM018_1 are presented as white and yellow letters, respectively. Note that the order of major and minor types is rearranged to one of their matching references (NC_003119 or HM159567).

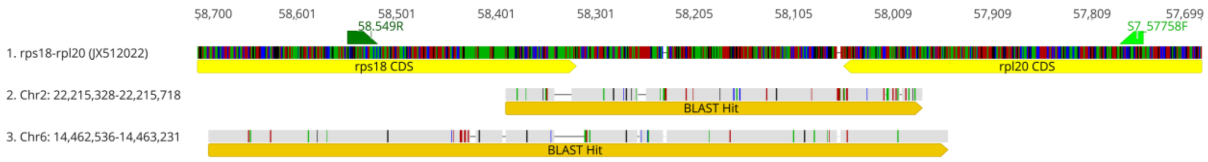

Figure S2. Sequence comparison among paralogous loci of the plastid *rps18-rpl20* region in the nuclear genome of R108 (GWHBFSB00000000) and the Jemalong 2HA (JX512022). Nucleotides in the reference sequence of JX512022 are presented as continuum of four different colored lines (Green = A, red = T, black = G, blue = C). Positions from the reference plastome are depicted above the sequence. The location of the plastid sequence in R108 nuclear genomes was presented with the chromosome (Chr) number on the left of the sequences.

Identical sequences from two sequences of the R108 nuclear genome, compared to the reference, are shown as grey. Annotations of primer, protein-coding sequence (CDS), and BLAST hit are marked in green, yellow, and brown, respectively.
